# Characteristics of Persistent Symptoms Manifested after SARS-CoV-2 Vaccination: An Observational Retrospective Study in a Specialized Clinic for Vaccination-Related Adverse Events

**DOI:** 10.3390/vaccines11111661

**Published:** 2023-10-30

**Authors:** Kazuki Tokumasu, Manami Fujita-Yamashita, Naruhiko Sunada, Yasue Sakurada, Koichiro Yamamoto, Yasuhiro Nakano, Yui Matsuda, Yuki Otsuka, Toru Hasegawa, Hideharu Hagiya, Hiroyuki Honda, Fumio Otsuka

**Affiliations:** 1 Department of General Medicine, Okayama University Graduate School of Medicine, Dentistry and Pharmaceutical Sciences, 2-5-1 Shikata-cho, Kitaku, Okayama 700-8558, Japan; tokumasu@okayama-u.ac.jp (K.T.); pdoi9n20@okayama-u.ac.jp (M.F.-Y.); pjbc9rog@s.okayama-u.ac.jp (N.S.); pzaf6h9w@s.okayama-u.ac.jp (Y.S.); pi291nd8@s.okayama-u.ac.jp (K.Y.); me421055@s.okayama-u.ac.jp (Y.N.); phvw0350@okayama-u.ac.jp (Y.M.); otsuka@s.okayama-u.ac.jp (Y.O.); pwcp0od9@s.okayama-u.ac.jp (T.H.); ppgf1hrd@okayama-u.ac.jp (H.H.); 2 Department of Infectious Diseases, Okayama University Hospital, Okayama 700-8558, Japan; hagiya@okayama-u.ac.jp

**Keywords:** adverse events, COVID-19, SARS-CoV-2 vaccination, mRNA vaccine

## Abstract

**Background:** Although many adverse reactions after SARS-CoV-2 vaccination have been reported, there have been few comprehensive studies on persistent symptoms after SARS-CoV-2 vaccination. The aim of this study was to determine the clinical characteristics of patients with various persistent symptoms after SARS-CoV-2 vaccination. **Methods:** A retrospective descriptive study was performed for patients who visited a specialized clinic established at Okayama University Hospital to evaluate adverse events after SARS-CoV-2 vaccination during the period from April 2021 to March 2023. **Results:** Descriptive analysis was performed for 121 of 127 patients who visited the clinic during the study period, and separate analysis was performed for the other 6 patients who had serious complications, who required treatment with prednisolone, and who had persistent symptoms. The median [interquartile range] age of the patients was 48 years [31–64 years], and the patients included 44 males (36.4%) and 77 females (63.6%). The most frequent symptoms were sensory impairment (34 patients, 28.1%), general fatigue (30 patients, 24.8%), fever/low-grade fever (21 patients, 17.4%), and headache (21 patients, 17.4%). Serious complications included myalgic encephalomyelitis/chronic fatigue syndrome (ME/CFS), sarcoidosis, aseptic meningitis, neuromyelitis optica spectrum disorders (NMOSDs), tendon adhesions, and idiopathic thrombocytopenia. **Conclusions:** Although causal relationships were not determined, 15 persistent symptoms after SARS-CoV-2 vaccination were characterized. All of the symptoms had onset from 12 hours to one week after vaccination, with 10 symptoms persisting for 6 months or longer. The most frequent symptom was sensory impairment.

## 1. Introduction

The emergence of the novel coronavirus disease 2019 (COVID-19) triggered a serious pandemic [1] and vaccines for SARS-CoV-2 were shown to be effective for preventing severe cases of acute COVID-19 [2,3,4]. In May 2023, WHO declared the end of the COVID-19 global health emergency and recommended a shift to persistent management of the disease [5,6]. However, the virus continues to infect millions of people worldwide each week along with the emergence of new Omicron subvariants, and its global risk remains high [5]. 

The development of mRNA vaccines for SARS-CoV-2 was one of the major turning points in the convergence process of the pandemic, with 13.3 billion doses given worldwide [6]. Currently, 89% of healthcare workers and 82% of adults over 60 years of age have globally received the primary course of a COVID-19 vaccine [6]. In addition to the prevention of infection, vaccinations have been shown to be effective for preventing severe cases of acute COVID-19 [6]. For recognition of the importance of vaccination against SARS-CoV-2 and for increasing immunization coverage, it is important to fully understand the possible adverse events related to mRNA vaccination. Previous studies have shown various vaccination-associated manifestations such as pain, swelling, and redness of the injection site as well as systemic reactions including fever, fatigue, myalgia, and headache [7,8,9,10,11]. 

Reports on serious complications after mRNA vaccination should also be noted. Fatal reactions including thrombotic thrombocytopenia, myocarditis/pericarditis, rhabdomyolysis, and anaphylaxis were noted in a previous review article [12]. Both central and peripheral nervous systems have also been reported to be involved in adverse reactions to mRNA vaccines [13]. Possible central nervous system disorders include venous sinus thrombosis, ischemic stroke, intracerebral hemorrhage, subarachnoid bleeding, reversible cerebral vasoconstriction syndrome, vasculitis, pituitary apoplexy, Susac syndrome, encephalitis, meningitis, demyelinating disorders, transverse myelitis, and epilepsy [13]. Peripheral nervous system disorders include Guillain–Barre syndrome, Parsonage–Turner syndrome, small fiber neuropathy, myasthenia, and myositis/dermatomyositis [13].

Despite continued efforts to uncover clinical uncertainties of mRNA vaccines, there is still insufficient information regarding the persistent symptoms that occur after vaccination. The aim of the present study was to determine the clinical characteristics of patients suffering from persistent symptoms after mRNA vaccination and to explore serious conditions based on our clinical experiences in a specialized clinic for vaccination-related adverse events.

## 2. Patients and Methods

### 2.1. Enrollment of Patients

This study was a descriptive study conducted in a single facility. A specialized outpatient clinic for managing patients suffering from persistent adverse events following SARS-CoV-2 vaccines was established in April 2021 in the Department of General Medicine of Okayama University Hospital, a tertiary hospital with 865 beds located in the western area of Japan. The establishment of the clinic was specifically commissioned by the Vaccine Strategy Office, Health and Medical Services Division, Department of Health and Medical Services, Okayama Prefectural Government. Patients were referred from outside medical facilities as well as other clinics in the university hospital. Patients who suffered from various symptoms for more than five days after SARS-CoV-2 vaccinations were enrolled in this study, based on a report that all reactions after vaccinations resolved within three to four days [7].

### 2.2. Collection of Clinical Data

Clinical information on patients who visited the specialized clinic to evaluate adverse events of SARS-CoV-2 vaccinations was obtained retrospectively. Medical records for 164 patients who visited the clinic during the period from April 2021 to March 2023 were carefully reviewed. Information on age, gender, body mass index (BMI), number of vaccinations, types of vaccination, and clinical symptoms was obtained from electric medical records. 

### 2.3. Definition of Serious Complications

Serious complications after SARS-CoV-2 vaccination were reported to be thrombotic thrombocytopenia, myocarditis/pericarditis, Guillain–Barre syndrome, acute transverse myelitis, and rhabdomyolysis [12]. We also included patients with serious complications who had severe inflammation, who needed treatment with prednisolone, or who had a difficult curable status with persistent symptoms.

### 2.4. Statistical Analyses

Descriptive statistical analyses were performed using Stata/SE 17.0 (StataCorp, 4905 Lakeway Dr, College Station, TX, USA). Differences in the frequencies of symptoms as possible adverse events between two groups were compared using Fisher’s exact test or Pearson’s χ2 test for categorical variables. A *p* value of less than 0.05 was considered statistically significant.

### 2.5. Ethical Approval

Information regarding the present study was provided on the website of our hospital, and patients who wished to opt out were offered that opportunity. Informed consent from the patients was not necessary due to the anonymization of data. This study was approved by the Ethics Committee of Okayama University Hospital (No. 2208-061) and adhered to the Declaration of Helsinki.

## 3. Results

Of the 164 patients who visited the specialized clinic to evaluate adverse events after SARS-CoV-2 vaccination during the study period, we excluded 37 patients because they visited the clinic in acute phases of adverse events (within 4 days after SARS-CoV-2 vaccinations). Data for 127 patients who visited our clinic were obtained, and descriptive analysis was performed for 121 patients. Detailed clinical courses of six patients with serious complications are also shown.

The clinical backgrounds of 121 patients with persistent symptoms after SARS-CoV-2 vaccinations are shown in Table 1. The median age of the patients was 48 years, without an apparent deviation in the age distribution. The patients included 44 males (36.4%) and 77 females (63.6%). The number (percentage) of patients who were referred from outside medical facilities was 106 (87.6%) and the number (percentage) of patients who were referred from other departments in the university hospital was 11 (9.1%). The administered timings of vaccines were actually varied, in which the receiving doses of vaccinations were as follows: one time: 32 cases (26.4%), two times: 37 cases (30.6%), three times: 35 cases (28.9%), and four times and more: 17 cases (14%). Most of the known vaccinations were mRNA vaccines provided by Pfizer (47.9%) or Moderna (9.9%), although the remaining cases (42.1%) were not clear on either of the mRNA vaccinations. 

**Figure 1:**
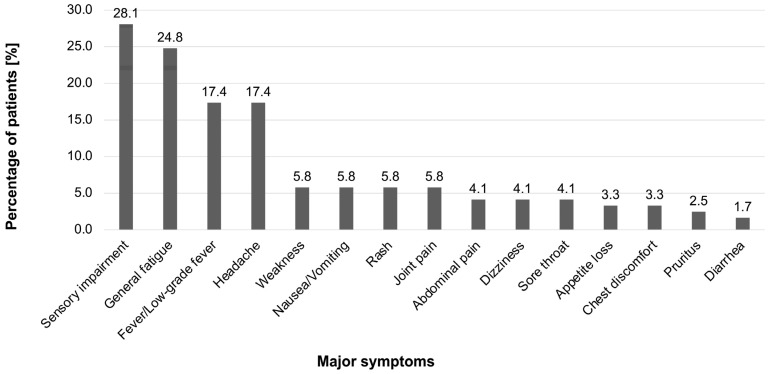
Persistent symptoms after SARS-CoV-2 vaccination classified by frequency. The four most frequent symptoms were sensory impairment (28.1% of the patients), general fatigue (24.8%), fever/low-grade fever (17.4%), and headache (17.4%). We included symptoms that were observed in more than two patients.

The percentages of persistent symptoms after vaccination are shown in Figure 1. The common symptoms in order of frequency were as follows: sensory impairment (34 patients, 28.1%), general fatigue (30 patients, 24.8%), fever/low-grade fever (21 patients, 17.4%), headache (21 patients, 17.4%), weakness (7 patients, 5.8%), nausea/vomiting (7 patients, 5.8%), rash (7 patients, 5.8%), joint pain (7 patients, 5.8%), abdominal pain (5 patients, 4.1%), dizziness (5 patients, 4.1%), sore throat (5 patients, 4.1%), appetite loss (4 patients, 3.3%), chest discomfort (4 patients, 3.3%), pruritus (3 patients, 2.5%), and diarrhea (2 patients, 1.7%). The regions of sensory impairment are shown in Table 2 (*n =* 37). The unilateral upper limb was the most frequent region (10 patients, 27.0%) for sensory impairment. Sensory impairment included numbness, dysesthesia, paresthesia, and sensory loss in not only the extremities but also the face, head, lips, tongue, and whole body.

**Figure 2:**
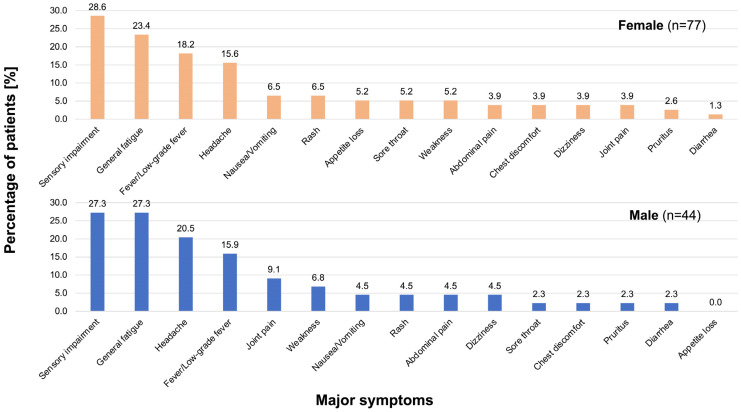
Gender-separated persistent symptoms after SARS-CoV-2 vaccination. The upper panel shows the percentage of symptoms in female patients (*n =* 77) and the lower panel shows the percentage of symptoms in male patients (*n =* 44). There were no significant differences between the two groups.

Gender-specific symptoms are shown in Figure 2. The proportions of patients with the two most frequent symptoms were almost the same in both genders (female vs. male): sensory impairment (28.6% vs. 27.3%) and general fatigue (23.4% vs. 27.3%). The percentages of patients with the third and fourth most frequent manifestations (fever/low-grade fever and headache) were also similar. There were no statistically significant differences in symptoms between the gender groups.

**Figure 3:**
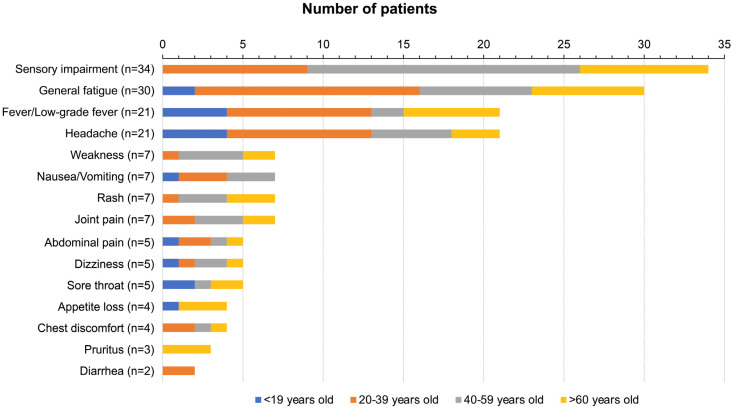
Age-related persistent symptoms after SARS-CoV-2 vaccination. The blue bar, orange bar, gray bar, and yellow bar indicate less than 19 years old, 20–39 years old, 40–59 years old, and over 60 years old, respectively.

The numbers of patients with each symptom in age groups (<19 years, 20–39 years, 40–59 years, >60 years) are shown in Figure 3. Sensory impairment was the most frequent symptom in the 40–59 years age group, and general fatigue, fever/low-grade fever, and headache were the most frequent symptoms in the 20–39 years age group. The numbers of patients with weakness, nausea/vomiting, rash, and joint pain were higher in the 40–59 years age group, though the numbers of patients were still quite small.

**Figure 4:**
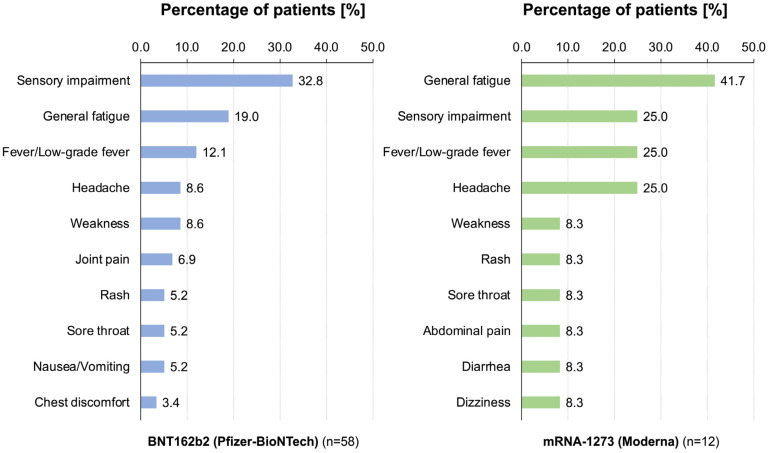
Types of vaccine-related persistent symptoms after SARS-CoV-2 vaccination. The panel on the left side shows the percentage of patients with each symptom among patients who received BNT162b2 (Pfizer-BioNTech) (*n =* 58) and the panel on the right side shows the percentage of patients with each symptom among patients who received mRNA-1273 (Moderna) (*n =* 12).

The types of vaccine-related persistent symptoms after SARS-CoV-2 vaccination are shown in Figure 4. The panel on the left side shows the percentage of patients with each symptom among patients who received BNT162b2 (Pfizer-BioNTech) (*n =* 58) and the panel on the right side shows the percentage of patients with each symptom among patients who received mRNA-1273 (Moderna) (*n =* 12). The most frequent persistent symptom caused by the BNT162b2 vaccine was sensory impairment (32.8%) as also shown in Figure 1. On the other hand, general fatigue (41.7%) was the most frequent persistent symptom in patients who received the mRNA-1273 vaccine.

**Figure 5:**
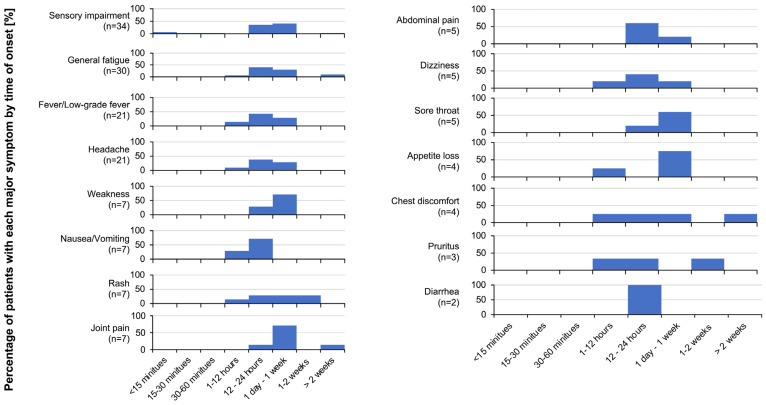
Timings of the onset of persistent symptoms after SARS-CoV-2 vaccination. The diagram shows when each of the symptoms occurred after vaccination.

The timings of the onset of reactive symptoms after vaccination are shown in Figure 5. Most of the symptoms occurred between one hour and one week after vaccination. The timings of the onset of persistent symptoms of sensory impairment and general fatigue, which were the most frequent symptoms, were 12 h to 1 week after vaccination, with all patients complaining of sensory impairment occurring within that period. In some cases, joint pain and chest discomfort occurred more than two weeks after vaccination.

**Figure 6:**
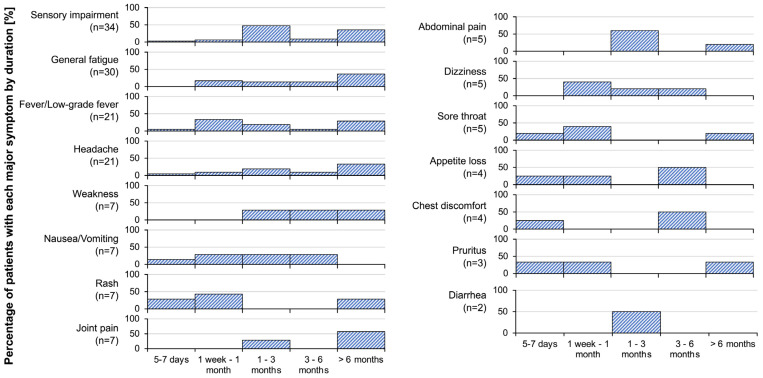
Durations of symptoms after SARS-CoV-2 vaccination. The diagram shows how long each of the symptoms continued after vaccination.

The durations of symptoms are shown in Figure 6. The durations of symptoms varied, but many symptoms persisted for more than 6 months. Symptoms that persisted for more than 6 months were sensory impairment, general fatigue, fever/low-grade fever, headache, weakness, rash, joint pain, abdominal pain, sore throat, and pruritus. Sensory impairment, which was the most frequent symptom, persisted for 1–3 months and more than 6 months in some patients, and general fatigue, the second most frequent symptom, also persisted for more than 6 months.

A case series of serious complications after SARS-CoV-2 vaccination is described below. A summary of these cases is shown in Table 3.

**Case 1:** Myalgic encephalomyelitis/chronic fatigue syndrome (ME/CFS). A 41-year-old woman was referred to our clinic by her family doctor with complaints of headache, dizziness and nausea, which started 1 h after the first vaccination (BNT162b2; Pfizer-BioNTech). The patient was admitted for exclusion of any possible underlying disorders. Blood examinations, thoracoabdominal CT, and head magnetic resonance imaging (MRI) showed no obvious abnormality. Rheumatological, endocrinological, and psychological diseases were also excluded through further investigations. The patient was treated symptomatically but developed fatigue and generalized pain after discharge. Even after six months, the patient continued to experience general fatigue and post-exertional malaise as well as persistent dizziness from standing up fast, poor concentration, and sleeping disorders. She also had swollen right cervical lymph nodes. She was able to undertake light work on good days; however, she needed to rest at home for more than half of the week. For these reasons, the patient was diagnosed with ME/CFS according to the Fukuda criteria, Canadian Consensus Criteria, and the Institute of Medicine (IOM) criteria. The patient was treated with ascorbic acids, ubidecarenone, ramelteon, and antiemetic medication for prolonged symptoms, and her general fatigue gradually improved. 

**Case 2:** Sarcoidosis. A 33-year-old man had a history of hormone treatment and surgery for gender identity disorder (female to male). After the second BNT162b2 (Pfizer-BioNTech) vaccination, he had persistent low-grade fever and malaise as well as foggy vision and hyperemia. The patient was diagnosed with uveitis by an ophthalmologist and was referred to our hospital and hospitalized for systemic examinations. A chest CT scan showed lymphadenopathy in his bilateral hilar regions and a granular shadow in his right lung despite no elevation of serum angiotensin-converting enzyme (ACE) or lysozyme. Gallium scintigraphy showed accumulation in his parotid gland and lacrimal gland and hilar lymphadenopathy. A biopsy of the right parotid gland showed noncaseating granulomas, leading to a pathological diagnosis of sarcoidosis. The patient was administered topical 0.1% betamethasone ophthalmic drops and oral prednisolone at 20 mg daily. He subsequently had a good clinical course with tapering prednisolone.

**Case 3:** Post-vaccination aseptic meningitis. An 84-year-old woman with a history of urticaria lasting for 2 months after the second COVID-19 vaccination (BNT162b2; Pfizer-BioNTech) developed fever and headache 6 days after the third COVID-19 vaccination (mRNA-1273; Moderna). Three weeks after the third vaccine, she was referred to our hospital because of hypoxia. She was conscious and showed no signs of meningeal irritation. Laboratory workup showed elevations of serum levels of C-reactive protein (19.6 mg/dL) and creatinine (2.1 mg/dL) and decreased serum levels of albumin (1.6 g/dL). Cerebrospinal fluid was clear and watery with normal pressure (160 mmH_2_O), but an elevated cell count (170/µL), low glucose (55 mg/dL), and elevated protein (67 mg/dL) were observed. The results of bacterial culture of cerebrospinal fluid were negative. Head MRI showed no abnormal findings indicating encephalitis or meningitis. Since intravenous antibiotic therapy was ineffective, high-dose steroid therapy was started with a diagnosis of vaccine-associated aseptic meningitis, and her symptoms rapidly resolved.

**Case 4:** Neuromyelitis optica with positive anti-aquaporin 4 antibody. A 74-year-old man with a medical history of hypertension developed lower back pain shortly after mRNA COVID-19 vaccination (mRNA-1273; Moderna) eight weeks ago. The patient was referred to us for investigation of headaches, fever, and anorexia that had persisted for the past three weeks. Physical assessment revealed neck stiffness, and the results of a lumbar puncture showed an elevated level of total protein (63 mg/dL) and elevated cell count (76/μL; monocytes: 100%) with a normal level of glucose (47 mg/dL). Treatments with anti-tuberculosis drugs and antifungal drugs were ineffective. Subsequently, he developed urinary retention and paralysis and sensory disturbance in both lower extremities. Since gadolinium-enhanced lumbar spine MRI revealed longitudinal myelitis, neurologists were consulted for further examination and treatment. With a positive result for serum anti-aquaporin (AQP) 4 antibody, he was diagnosed with neuromyelitis optica spectrum disorders (NMOSDs). After a combination of plasma exchange therapy, steroid pulse therapy, and intravenous immunoglobulin therapy, his neuropathy symptoms gradually improved, but immunosuppressive therapy was continued with rehabilitation.

**Case 5:** Tendon adhesions. The patient was a 48-year-old man who was healthy by nature. Ten days after the third vaccination (mRNA-1273; Moderna), he became immobile due to generalized pain. His local physician only found a slightly elevated level of C-reactive protein (2 mg/dL) and he was treated symptomatically. However, pain in his bilateral hip joints and proximal muscles of his limbs, which was worsened by exercise and was accompanied by limitation of movement and rest stiffness, did not improve. He was referred to our clinic and admitted to our hospital for further examination. Enhanced MRI revealed inflammation of hip adhesion; however, no specific collagen or infectious disease including spondylarthritis or psoriasis was identified using serological and imaging examinations. The patient was administered 10 mg per day of prednisolone with a diagnosis of idiopathic tendon adhesions, and prednisolone was tapered by 1 mg per month after discharge. 

**Case 6:** Idiopathic thrombocytopenia (ITP). An 81-year-old man who underwent oral surgery and chemotherapy (dacarbazine and interferon-beta) for right-sided maxillary malignant melanoma received his first dose of COVID-19 vaccine two days before a visit (vaccine type unknown). On a routine visit to the oral surgery department, he was found to have marked thrombocytopenia (platelet count of 4000/µL) and was referred to our clinic for examination and treatment. The patient had mucosal hemorrhage and subcutaneous hemorrhage and was thus urgently admitted to our hospital. We initiated 20 units of platelet transfusion along with prednisolone and immunoglobulin therapy. His platelet count did not increase in the first few days but then gradually increased. His bleeding symptoms also disappeared, and the patient was discharged home on the 18th day of hospitalization. Doses of prednisolone were tapered down to discontinuation after 5 months. Systemic investigations did not lead to a diagnosis of secondary thrombocytopenia, but platelet-associated immunoglobulin (PA-IgG) was found to be positive (3190 ng/10^7^ cells), leading to the diagnosis of ITP.

## 4. Discussion

In the present study, we determined the proportions of patients with persistent symptoms and the characteristics of persistent symptoms that occurred after SARS-CoV-2 vaccination based on results of examinations in our specialized clinic established for the patients with vaccination-related adverse events. The proportion of female patients in our study was 1.7 times higher than that of male patients (63.6% vs. 36.4%). The large proportion of female patients in our study is in agreement with the results of a population-based study showing that the incidence of neurological autoimmune diseases following vaccinations against SARS-CoV-2 was 3.2 times higher in women in Germany [14]. Underlying factors in gender differences might be involved in the female predominance in vaccination-related adverse events. Some studies have shown more adverse drug reactions in women [15] and differences in social determinants due to gender [16]. Furthermore, hemodynamics due to differences in body size may differ.

In the present study, sensory impairment was the most frequent symptom (in 34 (28.1%) of the 121 patients). Several studies have shown neurological symptoms after SARS-CoV-2 vaccination [13,14,17,18,19,20]. The reported neurological symptoms include both central nervous system disorders, such as headache, venous sinus thrombosis, ischemic stroke, intracerebral hemorrhage, subarachnoid bleeding, reversible cerebral vasoconstriction syndrome, vasculitis, pituitary apoplexy, Susac syndrome, encephalitis, meningitis, demyelinating disorders, transverse myelitis, and epilepsy, and peripheral nerve disorders (Guillain–Barre syndrome), Parsonage–Turner syndrome (plexitis), small fiber neuropathy, myasthenia, myositis/dermatomyositis, and rhabdomyolysis have been reported [13,14,17,18,19,20]. 

As shown in Figure 5, sensory impairment was the most frequent symptom persisting for 1–3 months followed by symptoms that persisted for 6 months or more. There is a case report of a 52-year-old woman who developed sensory disturbance from day 12 after her second dose of AstraZeneca vaccine with symptoms persisting for at least 7 months [21]. Various hypotheses regarding the SARS-CoV-2 vaccine and causes of these symptoms have been proposed [13]. Vaccine-induced S-protein or some of its peptide fragments not only stimulate the immune system but also bind to angiotensin-converting enzyme-2 (ACE-2) receptors on several types of cells surrounding the capillary bed as well as endothelial cells [22]. Via this mechanism, the S-protein enters cells and stimulates a series of intracellular responses that mimic SARS-CoV-2 infection [22]. The abundance of ACE-2 receptors on the cell surfaces is also responsible for the adverse effects of induction of inflammatory reactions by the nanoparticles used for mRNA delivery (PEGylation) [13]. Other hypotheses suggested that autoantibodies against platelet factor 4 and elevated D-dimer levels might be related to the neurological complications [23] and that inflammatory cytokines (interferons, interleukin (IL)-1-β, IL-6, and tumor necrosis factor) might cause headache after vaccination [24,25].

### Serious Complications after SARS-CoV-2 Vaccination

A principal researcher (K.T.) and co-researchers confirmed cases with serious complications that had severe inflammation, required the use of prednisolone, or had a difficult curable status with persistent symptoms. We included six cases according to the criteria. 

**Case 1:** ME/CFS. The patient met the three internationally standardized sets of ME/CFS criteria: the Fukuda criteria [26], Canadian Consensus Criteria [27], and IOM criteria [28]. There is no specific case report of a diagnosis of ME/CFS caused by SARS-CoV-2 vaccination, but there is a similar case report of long post-COVID vaccination syndrome [29]. In this case, a 39-year-old patient had persistent symptoms after the third mRNA-1273 (Moderna) vaccine. The patient’s symptoms included polyarthralgia, cognitive impairment, headache, insomnia, and staggering vertigo/dizziness [29], which are important components of ME/CFS’s diagnostic criteria.

**Case 2:** Sarcoidosis. This case is described as a case report with a literature review according to COVID-19 mRNA vaccine-associated uveitis leading to a diagnosis of sarcoidosis [30]. That case report also noted three other cases of pathogenically proved sarcoidosis or Lofgren syndrome [31,32]. There was also another case report of drug-induced sarcoidosis-like reaction after SARS-CoV-2 vaccination [33]. Thus, consideration should be given to whether sarcoidosis-like manifestations are caused by a drug or not.

**Case 3:** Post-vaccination aseptic meningitis. Aseptic meningitis after vaccinations for mumps, rubella, varicella-zoster virus, and others has been reported and it has been shown that measles, mumps, and rubella (MMR) vaccines cause meningitis in 1 case in 10,000 to 15,000 cases [34]. An immunological mechanism due to type III or IV hypersensitivity reactions is assumed to be the pathogenesis [35]. Various neurological complications have been reported with COVID-19 vaccines, including several cases of aseptic meningitis [12]. Almost all of the cases of aseptic meningitis caused by a COVID-19 vaccine had fever and headache, and the symptoms improved rapidly with steroid administration in many cases [36,37,38].

**Case 4:** Neuromyelitis optica with positive anti-aquaporin 4 antibody. NMOSD is an autoimmune disease that causes demyelination of the central nervous system including the optic nerve and the spinal cord. It was suggested that COVID-19 vaccinations are associated with the development of NMOSD in a systematic review of studies in which 26 patients experienced new demyelinating symptoms related to NMOSD [39]. In those patients, 54% (14/26) of the patients received an mRNA vaccine, 31% (8/26) of the patients received a viral vector vaccine, and 15% (4/26) of the patients received an inactivated COVID-19 vaccine [39]. There was a female preponderance (female/male: 3.3/1), and the median age of the patients was 50 years (range: 19–80 years) [40]. NMO symptoms appeared within a median interval of 10 days (range: 1–97 days) after the COVID-19 vaccination [39]. As shown in our case, NMOSD might be one of the important disorders that develop after COVID-19 vaccination.

**Case 5:** Tendon adhesions. There is no case report of an apparent association between tendon adhesions and SARS-CoV-2 vaccination. Tendon adhesions are often associated with spondyloarthritis and psoriasis, but there is also no report of these diseases occurring after vaccination. The common disease that causes generalized pain in major joints is rheumatoid arthritis (RA), though there have been cases of RA following vaccination [40]. A 53-year-old healthy Japanese man had a swollen left knee joint four weeks after vaccination with BNT162b2 [40]. He also noticed morning stiffness. The serum level of anti-cyclic citrullinated peptide antibody was 1200 U/mL and magnetic resonance imaging showed diffuse knee joint effusion. Based on these typical symptoms and results of serological analysis, he was diagnosed with rheumatoid arthritis [40]. On the other hand, a study of 5493 RA patients in Hong Kong showed no significant association between arthritis flare-ups and COVID-19 vaccination [41]. 

**Case 6:** Idiopathic thrombocytopenia (ITP). There have been several reports of thrombocytopenia after vaccination, which may involve molecular mimicry in which pathogenic antibodies bind to platelets and megakaryocytes, causing thrombocytopenia via opsonization, complement activation, and apoptotic pathways [42].

The present study has several limitations. First, this study was carried out to determine the prevalence of persistent adverse reactions after receiving SARS-CoV-2 vaccinations in patients who visited a single outpatient clinic in Japan. Second, patients presenting with characteristic organ symptoms (i.e., hemiplegia) directly consulted a specialized department and were therefore not included in this study (i.e., patients with hemiplegia consult a neurologist.). Third, the present study was retrospective observational research with reference to medical records, and 51 (42.1%) of the participants were not able to clarify whether the vaccine they received was BNT162b2 or mRNA-1273. Finally, this study only showed adverse symptoms after vaccinations, and a causal relationship between vaccination and manifested symptoms could therefore not be clarified.

Collectively, our results showed the clinical characteristics of persistent symptoms after SARS-CoV-2 vaccination. The four major symptoms were sensory impairment (28.1% of the patients), general fatigue (24.8%), fever/low-grade fever (17.4%), and headache (17.4%). Other symptoms included weakness, nausea/vomiting, rash, joint pain, abdominal pain, dizziness, sore throat, appetite loss, chest discomfort, pruritus, and diarrhea. Adverse events following vaccination included a wide variety of symptoms, and physicians therefore need to consider all of the possible events in relation to vaccination, carefully and holistically, by encompassing various symptoms derived from each individual case.

## Figures and Tables

**Table 1:** Backgrounds of patients with persistent symptoms after SARS-CoV-2 vaccination.

Patients’ Backgrounds	Case Number (Total 121 Patients)
**Age**	
Median [IQR], years	48 [31–64]
<19 years	11 (9.1%)
20–39 years	31 (25.6%)
40–59 years	43 (35.5%)
>60 years	36 (29.8%)
**Gender**	
Male	44 (36.4%)
Female	77 (63.6%)
**BMI**	
Median [IQR]	22.6 [20.3–25.5]
**Source of referral**	
Outside medical facilities (clinics and hospitals)	106 (87.6%)
Other departments of in the university hospital	11 (9.1%)
unrecorded	4 (3.3%)
**Medical history**	
Hypertension	21 (17.4%)
Hyperlipidemia	21 (17.4%)
Diabetes mellites	7 (5.8%)
Autoimmune disease	11 (9.1%)
Malignancy	8 (6.6%)
Previous infection of SARS-CoV-2	6 (5.0%)
**Receiving times of causal SARS-CoV-2 vaccines**	
**First dose (*n =* 32)**	
1 April 2021–30 September 2021	25 (78.1%)
1 October 2021–	6 (18.8%)
unrecorded	1 (3.1%)
**Second dose (*n =* 37)**	
1 April 2021–30 September 2021	26 (70.3%)
1 October 2021–	11 (29.7)
**Third dose (*n =* 35)**	
1 October 2021–31 March 2022	14 (40%)
1 April 2022–	20 (57.1%)
Unrecorded	1 (2.9%)
**Fourth dose (*n =* 14)**	
1 June 2022–	12 (85.7%)
unrecorded	2 (14.3%)
**Fifth dose (*n =* 3)**	
1 November 2022–	3 (100%)
**Types of SARS-CoV-2 vaccines (*n =* 121)**	
BNT162b2 (Pfizer-BioNTech; New York City, NY, USA and Mainz, Germany)	58 (47.9%)
mRNA-1273 (Moderna; Cambridge, MA, USA)	12 (9.9%)
Unidentified	51 (42.1%)

Medians [IQR: interquartile ranges] and percentages (%) are shown. BMI: body mass index. The likelihood of patients receiving AstraZeneca’s DNA vaccine is almost nil.

**Table 2:** Specific regions of sensory impairment after SARS-CoV-2 vaccination.

Regions	Case Number (%) (Total 37 Patients)
Upper limbs (unilateral)	10 (27.0)
All extremities	7 (18.9)
Maniphalanx (unilateral)	7 (18.9)
Face, head	3 (8.1)
Hemiplegia	2 (5.4)
Lower limbs (bilateral)	2 (5.4)
Maniphalanx (bilateral)	2 (5.4)
Whole body	2 (5.4)
Upper limbs (bilateral)	1 (2.7)
Lips, tongue	1 (2.7)

Three cases had two regions of sensory impairment.

**Table 3:** Summary of six serious cases after SARS-CoV-2 vaccination.

	Onset after SARS-CoV-2 Vaccination	Remarkable Results of Examinations	Diagnosis	Treatment	Prognosis
**Case 1**	One hour after the first dose of the vaccine (BNT162b2; Pfizer-BioNTech)	Positive Schellong test (ruled out rheumatological disease, endocrine disease, and chronic infections)	ME/CFS	Ascorbic acids, Ubidecarenone, Ramelteon	Symptoms mildly improving
**Case 2**	A few—about 10 h after the second dose of the vaccine (BNT162b2; Pfizer-BioNTech)	Lymphadenopathy in the hilar region and granular shadow of the right lung, gallium scintigraphy showed accumulation in the parotid gland, lacrimal gland, and hilar lymphadenopathy, and a right parotid biopsy showed granulomas	Sarcoidosis	Initial treatment with prednisolone at 20 mg/day	Tapering prednisolone
**Case 3**	Six days after the 3rd dose of the vaccine (mRNA-1273; Moderna)	Head MRI: age-related chronic ischemic changes CSF examination; cell count 170	Post-vaccination aseptic meningitis	Methylprednisolone at 500 mg/day for 3 days, and then prednisolone at 50 mg/day	Tapering prednisolone
**Case 4**	Five weeks after the vaccination (mRNA-1273; Moderna)	Spinal cord MRI: high signal at L1-L5T2W1, longitudinal myelitis-like, anti-AQP4 antibody > 40 U/mL	Neuromyelitis optica with positive anti-aquaporin 4 antibody	Methylprednisolone at 500 mg/day for 3 days, plasma exchange and IVIG	Continuing prednisolone at 5 mg/day, high degree of paralysis of both lower limbs, residual urinary incontinence
**Case 5**	10 days after third vaccination (mRNA-1273; Moderna)	CRP elevation, inflammation of the vicinity of the adductor pollicis major attachment of the bilateral psoas minor muscles	Tendon adhesions	Prednisolone at 10 mg/day	Tapered prednisolone and off
**Case 6**	Within two days (BNT162b2; Pfizer-BioNTech or mRNA-1273 Moderna)	Thrombocytopenia (platelet count, 4000/μL) and positive PA-IgG (3190 ng/10^7^ cells)	ITP	IVIG at 2 g/day for 5 days, prednisolone at 50 mg/day	Tapered prednisolone during five months and off

AQP, aquaporin; ITP, idiopathic thrombocytopenia; IVIG, intravenous immunoglobulins; ME/CFS, myalgic encephalomyelitis/chronic fatigue syndrome; MRI, magnetic resonance imaging; PA-IgG, platelet-associated immunoglobulin.

## Data Availability

Detailed data will be available if requested to the corresponding author.

## Abbreviations

Angiotensin-converting enzyme (ACE); aquaporin (AQP); body mass index (BMI); central nervous system (CNS); coronavirus disease 2019 (COVID-19); Institute of Medicine (IOM); idiopathic thrombocytopenia (ITP); intravenous immunoglobulins (IVIG); myalgic encephalomyelitis/chronic fatigue syndrome (ME/CFS); magnetic resonance imaging (MRI); neuromyelitis optica spectrum disorder (NMOSD); World Health Organization (WHO).
